# The genome sequences of the marine diatom *Epithemia pelagica* strain UHM3201 (Schvarcz, Stancheva & Steward, 2022) and its nitrogen-fixing, endosymbiotic cyanobacterium

**DOI:** 10.12688/wellcomeopenres.21534.1

**Published:** 2024-04-29

**Authors:** Christopher R. Schvarcz, Rosalina Stancheva, Kendra A. Turk-Kubo, Samuel T. Wilson, Jonathan P. Zehr, Kyle F. Edwards, Grieg F. Steward, John M. Archibald, Graeme Oatley, Elizabeth Sinclair, Camilla Santos, Michael Paulini, Eerik Aunin, Noah Gettle, Haoyu Niu, Victoria McKenna, Rebecca O’Brien, Wellcome Sanger Institute Tree of Life Management, Samples and Laboratory Team, Wellcome Sanger Institute Scientific Operations: Sequencing Operations, Wellcome Sanger Institute Tree of Life Core Informatics Team, EBI Aquatic Symbiosis Genomics Data Portal Team, Aquatic Symbiosis Genomics Project Leadership

**Affiliations:** 1 Department of Oceanography, Daniel K. Inouye Center for Microbial Oceanography: Research and Education (C-MORE), University of Hawai'i at Manoa, Honolulu, Hawaii, USA; 2 Department of Environmental Science and Policy, George Mason University, Fairfax, Virginia, USA; 3 Department of Ocean Sciences, University of California Santa Cruz, Santa Cruz, California, USA; 4 School of Natural & Environmental Sciences, Newcastle University, Newcastle upon Tyne, England, UK; 5 Department of Biochemistry and Molecular Biology, Dalhousie University, Halifax, Nova Scotia, Canada; 6 Tree of Life, Wellcome Sanger Institute, Hinxton, England, UK

**Keywords:** Epithemia pelagica strain UHM3201, cyanobacterial endosymbiont, pennate diatom, genome sequence, chromosomal, Rhopalodiales

## Abstract

We present the genome assembly of the pennate diatom *Epithemia pelagica* strain UHM3201 (Ochrophyta; Bacillariophyceae; Rhopalodiales; Rhopalodiaceae) and that of its cyanobacterial endosymbiont (Chroococcales: Aphanothecaceae). The genome sequence of the diatom is 60.3 megabases in span, and the cyanobacterial genome has a length of 2.48 megabases. Most of the diatom nuclear genome assembly is scaffolded into 15 chromosomal pseudomolecules. The organelle genomes have also been assembled, with the mitochondrial genome 40.08 kilobases and the plastid genome 130.75 kilobases in length. A number of other prokaryote MAGs were also assembled.

## Species taxonomy: host

Eukaryota; Sar; Stramenopiles; Ochrophyta; Bacillariophyta; Bacillariophyceae; Bacillariophycidae; Rhopalodiales; Rhopalodiaceae; *Epithemia*; *Epithemia pelagica* strain UHM3201 (Schvarcz, Stancheva & Steward, 2022) (NCBI:txid2809013).

## Species taxonomy: endosymbiont

Bacteria; Terrabacteria group; Cyanobacteriota/Melainabacteria group; Cyanobacteriota; unclassified Cyanobacteriota; cyanobacterium endosymbiont of *Epithemia pelagica* strain UHM3201 (NCBI:txid2809053)

## Background

*Epithemia pelagica* is a single-celled marine pennate diatom belonging to the family Rhopalodiaceae. Similar to other species within this family ( Nakayama *et al.*, 2011; Prechtl *et al.*, 2004), *E. pelagica* hosts nitrogen-fixing cyanobacterial endosymbionts, which are thought to be in the early stages of becoming an organelle ( Kneip *et al.*, 2007; Nakayama *et al.*, 2014). *E. pelagica* was first isolated from open ocean waters north of Hawai‘i, in the North Pacific Ocean ( Schvarcz *et al.*, 2022). While this new diatom species has yet to be reported elsewhere, metagenomic analyses have detected gene sequences matching *E. pelagica*’s symbiont in tropical and subtropical marine habitats across the globe, suggesting this symbiosis is more widespread than currently reported.

*E. pelagica* is characterised by small, solitary cells measuring 6–18 µm long and 5–10 µm wide. Cells are strongly dorsiventral and asymmetrical along the apical axis, and valves are lunate with rounded apices, having a convex dorsal margin and concave ventral margin. *E. pelagica* differs from other species in the genus *Epithemia* by its minute size, weakly silicified frustules with delicate costae, and very fine striae that are not resolvable with light microscopy. *E. pelagica* cells typically harbor one or two endosymbionts, but cell cultures can lose their symbionts when grown for extended periods in nitrogen-rich medium. The endosymbionts lack fluorescent photosynthetic pigments and tend to be located next to the host cell’s nucleus.

The genome assemblies for *E. pelagica* and its endosymbiont will be a valuable resource for furthering our understanding of endosymbiosis and organellogenesis. These genomes will reveal the extent of endosymbiotic gene transfer to the diatom host and will guide future investigations of host-symbiont physiology, including the transfer of key metabolites. The genome of *E. pelagica* will also aid phylogenomic and evolutionary studies of the diatom order Rhopalodiales.

## Genome sequence report

**Figure 1.**
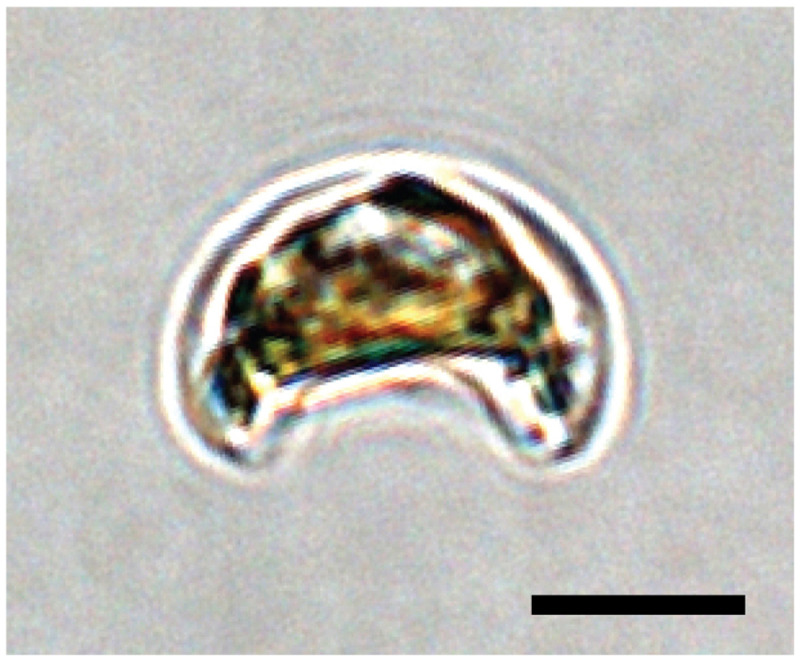
Light micrograph of a live *Epithemia pelagica* strain UHM3201 cell. Scale bar equals 5 µm.

The genome of *Epithemia pelagica* strain UHM3201 was sequenced from cultured cells ( Figure 1) isolated from seawater at Station ALOHA in the subtropical North Pacific Ocean ( Schvarcz *et al*., 2022). A total of 298-fold coverage in Pacific Biosciences single-molecule HiFi long reads was generated. Primary assembly contigs were scaffolded with chromosome conformation Hi-C data. Manual assembly curation corrected eight missing joins or mis-joins and removed one haplotypic duplication, reducing the scaffold number by 14.81%.

**Figure 2.**
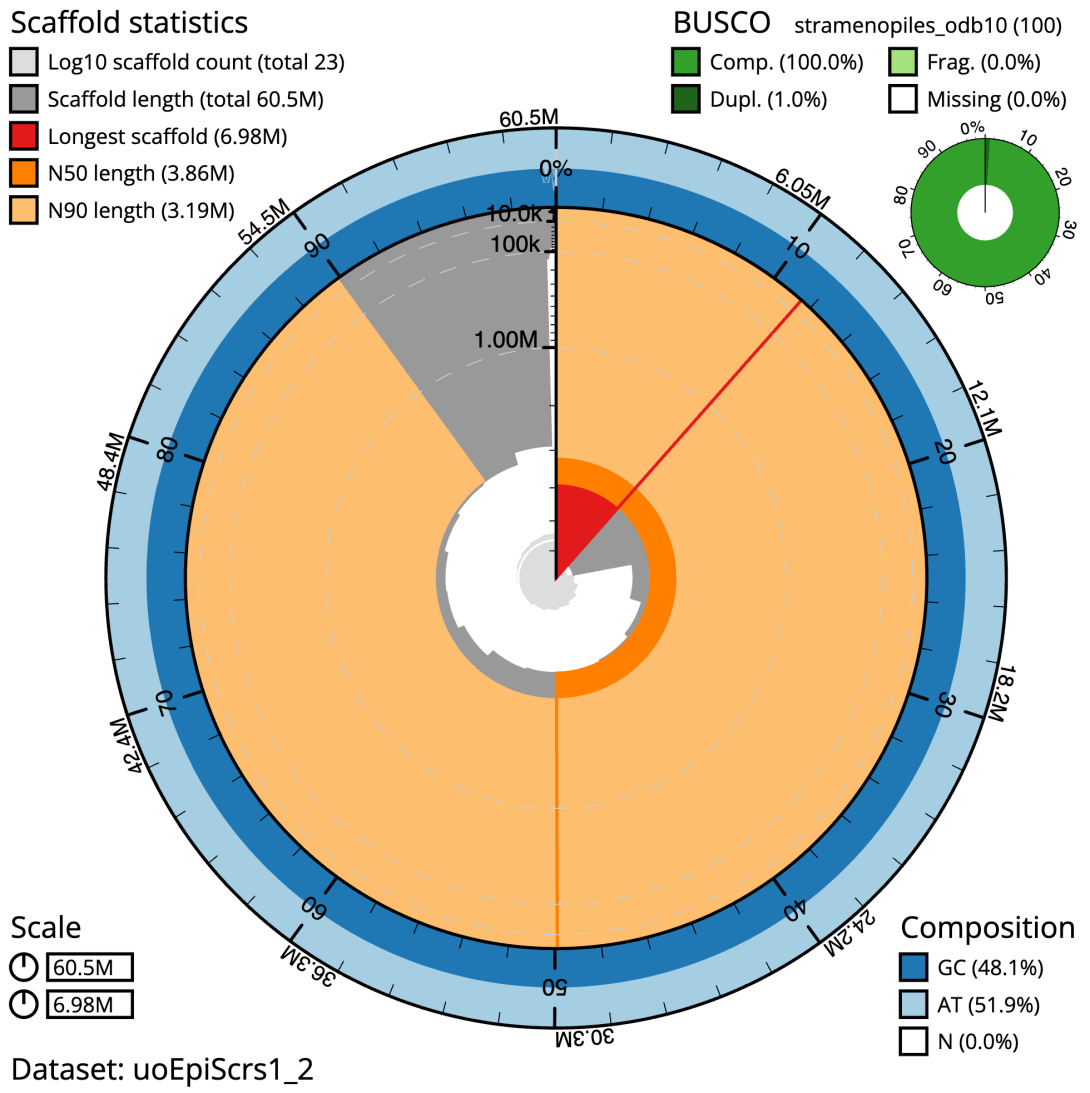
Genome assembly of *Epithemia pelagica*, uoEpiScrs1.2: metrics. The BlobToolKit snail plot shows N50 metrics and BUSCO gene completeness. The main plot is divided into 1,000 size-ordered bins around the circumference with each bin representing 0.1% of the 60,520,547 bp assembly. The distribution of scaffold lengths is shown in dark grey with the plot radius scaled to the longest scaffold present in the assembly (6,983,076 bp, shown in red). Orange and pale-orange arcs show the N50 and N90 scaffold lengths (3,856,736 and 3,186,670 bp), respectively. The pale grey spiral shows the cumulative scaffold count on a log scale with white scale lines showing successive orders of magnitude. The blue and pale-blue area around the outside of the plot shows the distribution of GC, AT and N percentages in the same bins as the inner plot. A summary of complete, fragmented, duplicated and missing BUSCO genes in the stramenopiles_odb10 set is shown in the top right. An interactive version of this figure is available at https://blobtoolkit.genomehubs.org/view/Epithemia%20pelagica/dataset/uoEpiScrs1_2/snail.

**Figure 3.**
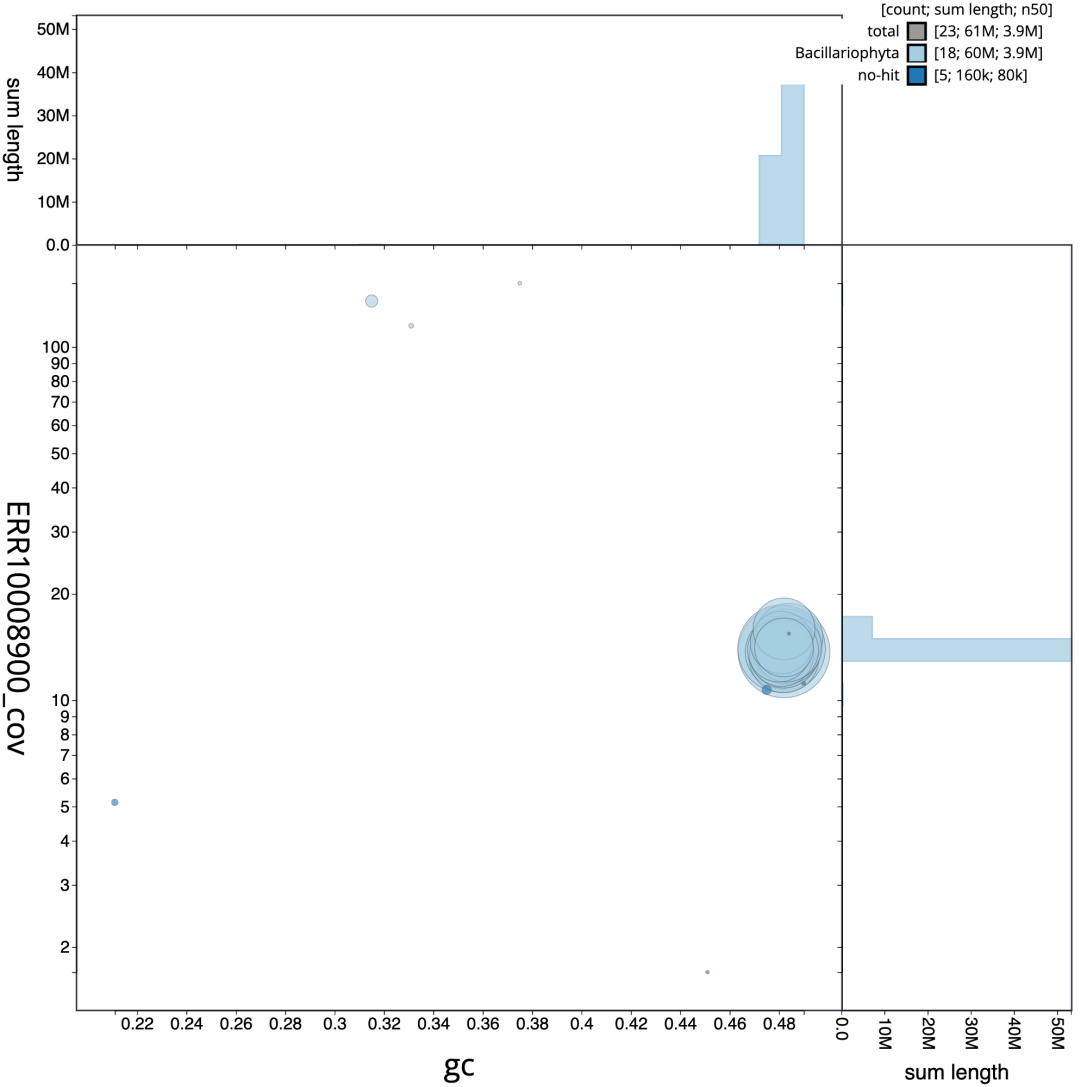
Genome assembly of *Epithemia pelagica*, uoEpiScrs1.2: BlobToolKit GC-coverage plot. Scaffolds are coloured by phylum. Circles are sized in proportion to scaffold length. Histograms show the distribution of scaffold length sum along each axis. An interactive version of this figure is available at https://blobtoolkit.genomehubs.org/view/Epithemia%20pelagica/dataset/uoEpiScrs1_2/blob.

**Figure 4.**
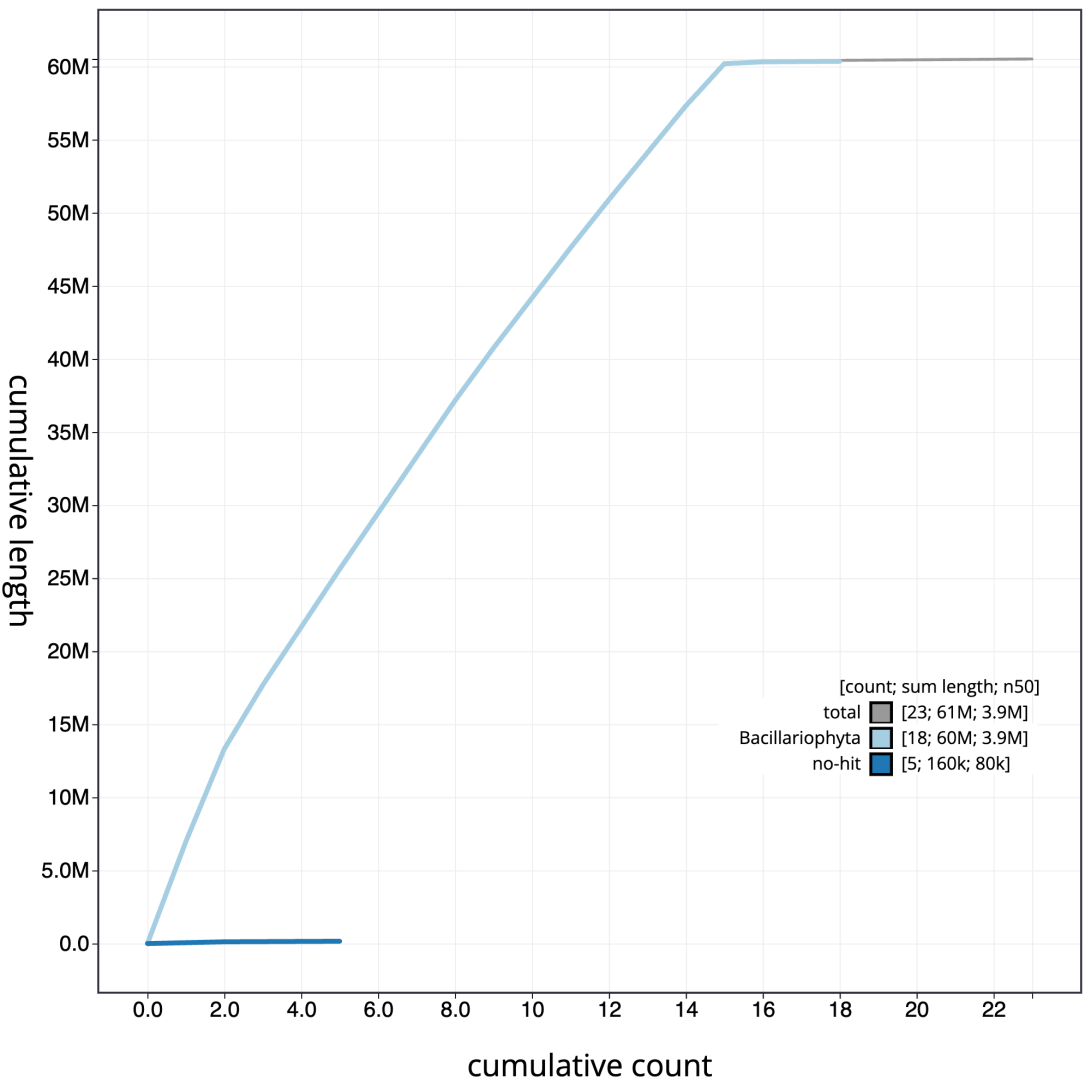
Genome assembly of *Epithemia pelagica*, uoEpiScrs1.2: BlobToolKit cumulative sequence plot. The grey line shows cumulative length for all scaffolds. Coloured lines show cumulative lengths of scaffolds assigned to each phylum using the buscogenes taxrule. An interactive version of this figure is available at https://blobtoolkit.genomehubs.org/view/Epithemia%20pelagica/dataset/uoEpiScrs1_2/cumulative.

**Figure 5.**
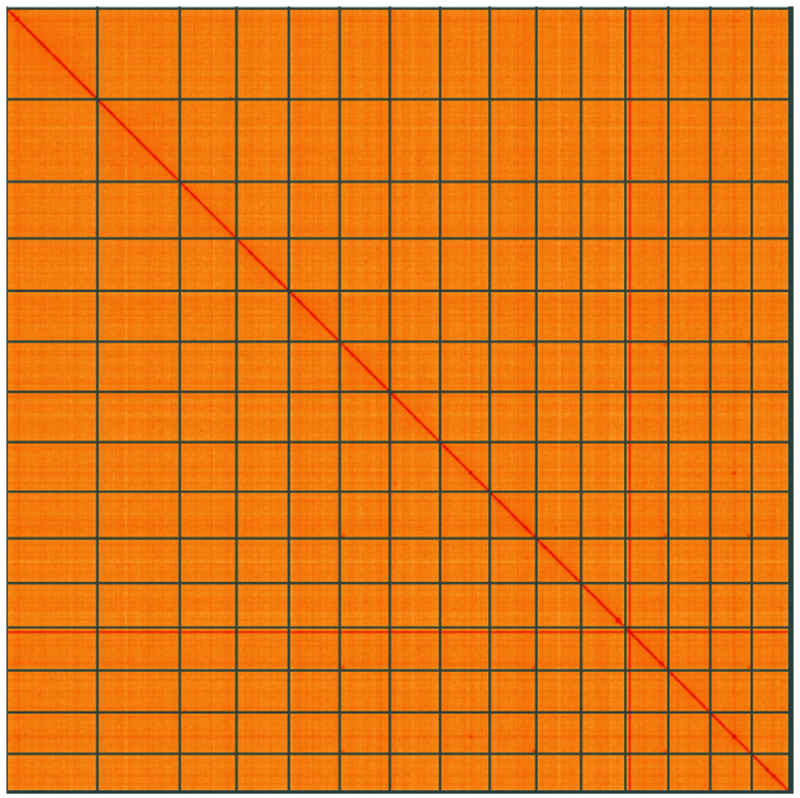
Genome assembly of *Epithemia pelagica*, uoEpiScrs1.2: Hi-C contact map of the uoEpiScrs1.2 assembly, visualised using HiGlass. Chromosomes are shown in order of size from left to right and top to bottom. An interactive version of this figure may be viewed at https://genome-note-higlass.tol.sanger.ac.uk/l/?d=ek4BBaV4Smikzb_1OOkKpQ.

The final assembly has a total length of 60.3 megabases (Mb) in 21 sequence scaffolds with a scaffold N50 of 3.9 Mb ( Table 1). The snail plot in Figure 2 provides a summary of the assembly statistics, while the distribution of assembly scaffolds on GC proportion and coverage is shown in Figure 3. The cumulative assembly plot in Figure 4 shows curves for subsets of scaffolds assigned to different phyla. Most (99.54%) of the assembly sequence was assigned to 15 chromosomal-level scaffolds. Chromosome-scale scaffolds confirmed by the Hi-C data are named in order of size ( Figure 5; Table 2). While not fully phased, the assembly deposited is of one haplotype. Contigs corresponding to the second haplotype have also been deposited. The mitochondrial and plastid genomes were also assembled (40.08 and 130.75 kilobases (kb) in size, respectively) and can be found as contigs within the multifasta file of the genome submission.

**Table 1.** Genome data for *Epithemia pelagica* strain UHM3201, uoEpiScrs1.2.

Project accession data		
Assembly identifier	uoEpiScrs1.2	
Species	*Epithemia pelagica* strain UHM3201	
Specimen	uoEpiScrs1	
NCBI taxonomy ID	2809013	
BioProject	PRJEB54946	
BioSample ID	SAMEA10835113	
Isolate information	uoEpiScrs1 cells (DNA, Hi-C)	
Assembly metrics *		*Benchmark*
Consensus quality (QV)	60.9	*≥ 50*
*k*-mer completeness	100%	*≥ 95%*
BUSCO **	C:100.0%[S:99.0%,D:1.0%], F:0.0%,M:0.0%,n:100	*C ≥ 95%*
Percentage of assembly mapped to chromosomes	99.54%	*≥ 95%*
Sex chromosomes	-	*localised homologous pairs*
Organelles	Mitochondrial and plastid genomes assembled	*complete single alleles*
Raw data accessions		
PacificBiosciences SEQUEL II	ERR10008900, ERR10008901	
Hi-C Illumina	ERR9988143	
Genome assembly		
Assembly accession	GCA_946965045.2	
*Accession of alternate haplotype*	GCA_946965055.2	
Span (Mb)	60.3	
Number of contigs	60	
Contig N50 length (Mb)	2.2	
Number of scaffolds	21	
Scaffold N50 length (Mb)	3.9	
Longest scaffold (Mb)	7.0	

* Assembly metric benchmarks are adapted from column VGP-2020 of “Table 1: Proposed standards and metrics for defining genome assembly quality” from ( Rhie *et al.*, 2021). ** BUSCO scores based on the stramenopiles_odb10 BUSCO set using v5.3.2. C = complete [S = single copy, D = duplicated], F = fragmented, M = missing, n = number of orthologues in comparison. A full set of BUSCO scores is available at https://blobtoolkit.genomehubs.org/view/Epithemia%20pelagica/dataset/uoEpiScrs1_2/busco.

**Table 2.** Chromosomal pseudomolecules in the genome assembly of *Epithemia pelagica*, uoEpiScrs1.

INSDC accession	Chromosome	Length (Mb)	GC%
OX337228.1	1	6.98	48.0
OX337229.1	2	6.34	48.0
OX337230.1	3	4.37	48.0
OX337231.1	4	4.03	48.0
OX337232.1	5	3.91	48.5
OX337233.1	6	3.86	48.0
OX337234.1	7	3.86	48.5
OX337235.1	8	3.81	48.5
OX337236.1	9	3.6	48.5
OX337237.1	10	3.43	48.0
OX337238.1	11	3.41	48.5
OX337239.1	12	3.31	48.0
OX337240.1	13	3.23	48.0
OX337241.1	14	3.19	48.0
OX337242.1	15	2.88	48.0
OX459761.1	Pltd	0.13	31.5
OX337243.1	MT	0.04	21.0

The estimated Quality Value (QV) of the final assembly is 60.9 with *k*-mer completeness of 100%, and the assembly has a BUSCO v5.3.2 completeness of 100% (single = 99.0%, duplicated = 1.0%), using the stramenopiles_odb10 reference set ( *n* = 100).

Metadata for specimens, barcode results, spectra estimates, sequencing runs, contaminants and pre-curation assembly statistics are given at https://links.tol.sanger.ac.uk/species/2809013.

**Figure 6.**
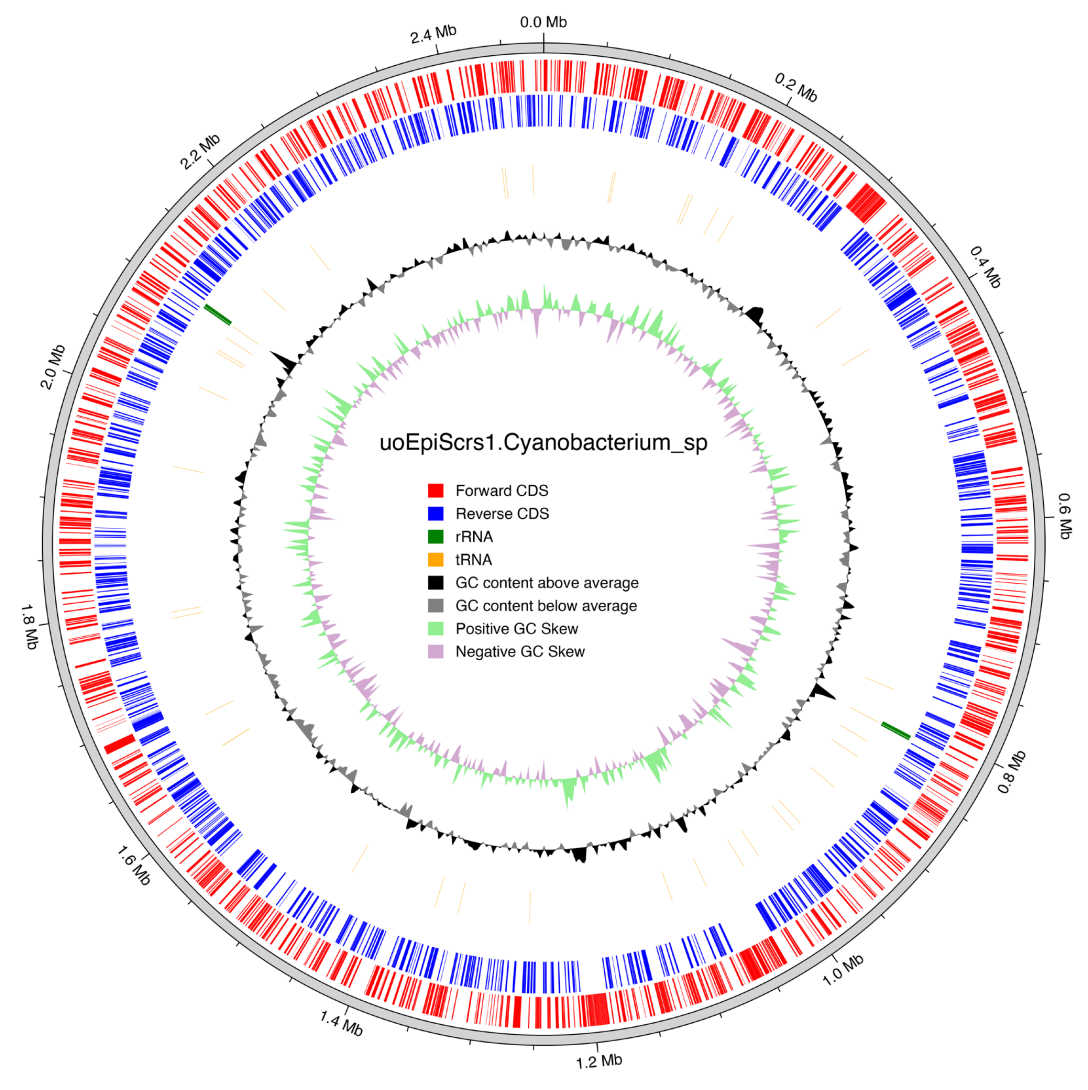
Genomic map of cyanobacterium endosymbiont of *Epithemia pelagica* strain UHM3201 (GCA_947331815.1). The tracks showing predicted coding regions, predicted tRNA and rRNA, and GC content. The Genbank file https://www.ncbi.nlm.nih.gov/datasets/gene/GCF_947331815.1/ was used to produce the map.

The genome of the cyanobacterial endosymbiont of *E. pelagica* (Chroococcales: Aphanothecaceae) was manually assembled and found to be 2.48 Mb in size ( Table 3, Figure 6). This novel species was named cyanobacterium endosymbiont of *Epithemia pelagica* strain UHM3201 (taxon ID: 2809053).

**Table 3.** Genome data for Cyanobacterium endosymbiont of *Epithemia pelagica* strain UHM3201.

Project accession data	
Assembly identifier	uoEpiScrs1.Cyanobacterium_sp_1.1
Species	Cyanobacterium endosymbiont of *Epithemia pelagica* strain UHM3201
NCBI taxonomy ID	2809053
BioProject	PRJEB54946
BioSample ID	SAMEA10835113 SAMEA111323721
Raw data accessions	
PacificBiosciences SEQUEL II	ERR10008900, ERR10008901
Hi-C Illumina	ERR9988143
Genome assembly	
Assembly accession	GCA_947331815.1
Span (Mb)	2.5

**Figure 7.**
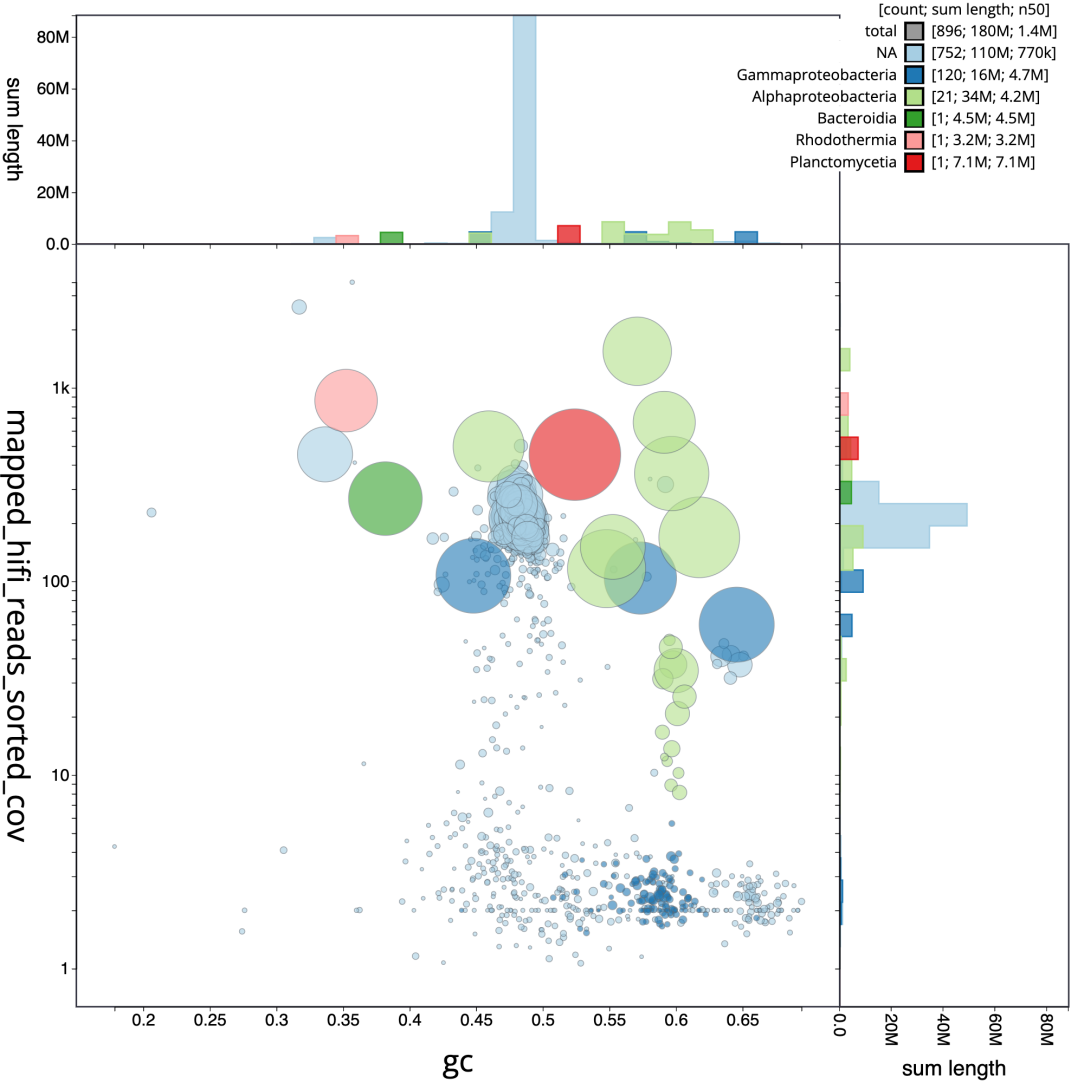
Metagenome of *Epithemia pelagica* strain UHM3201. Blob plot of mapped base coverage against GC proportion metaMDBG assembled contigs. Contigs are coloured by assigned taxonomic class where NA represents unbinned and eukaryotic sequences. Circles are sized in proportion to length on a square-root scale, ranging from 894 to 7,080,000. The assembly has been filtered to exclude records with mapped base coverages < 1. Histograms show the distribution of record length sum along each axis.

The metagenome of *E. pelagica* was assembled and MAGs belonging to the taxa *Erythrobacter* sp., *Ekhidna* sp., Pseudomonadales, Alphaproteobacteria, Thalassobaculaceae, *Alteromonas macleodii*, Pseudomonadales, Balneolaceae, *Aureliella* sp., *Thalassovita* sp., *Lentilitoribacter* sp., *Thalassovita* sp., *Dinoroseobacte*r sp., *Alcanivorax* sp., *Dinoroseobacter* sp. and *Marinobacter alexandrii* were identified ( Figure 7).

## Methods

### Sample acquisition and nucleic acid extraction

A sample of *Epithemia pelagica* (specimen ID DU0000022, ToLID uoEpiScrs1) was obtained from cultured cells ( Figure 1) isolated from seawater at Station ALOHA in the subtropical North Pacific Ocean. The cells were collected, and the diatom species was identified by Christopher Schvarcz (University of Hawai‘i at Mānoa), Rosalina Stancheva (George Mason University), and Grieg Steward (University of Hawai‘i at Mānoa). Cell pellets were collected by centrifugation (4,000 × *g* for 10 min), followed by transfer to a cryovial, and then snap-frozen in liquid nitrogen.

High molecular weight (HMW) DNA was extracted at the Tree of Life laboratory, Wellcome Sanger Institute (WSI), following a sequence of core procedures: sample preparation; sample homogenisation; HMW DNA extraction; DNA fragmentation; and DNA clean-up. The uoEpiScrs1 sample was prepared on dry ice ( Jay *et al.*, 2023). The cells were cryogenically disrupted using the Covaris cryoPREP ^®^ Automated Dry Pulverizer ( Narváez-Gómez *et al.*, 2023). HMW DNA was extracted using the Manual MagAttract v1 protocol ( Strickland *et al.*, 2023b). DNA was sheared into an average fragment size of 12–20 kb in a Megaruptor 3 system with speed setting 30 ( Todorovic *et al.*, 2023). Sheared DNA was purified by solid-phase reversible immobilisation ( Strickland *et al.*, 2023a): in brief, the method employs a 1.8X ratio of AMPure PB beads to sample to eliminate shorter fragments and concentrate the DNA. The concentration of the sheared and purified DNA was assessed using a Nanodrop spectrophotometer and Qubit Fluorometer and Qubit dsDNA High Sensitivity Assay kit. Fragment size distribution was evaluated by running the sample on the FemtoPulse system.

Protocols developed by the WSI Tree of Life laboratory are publicly available on protocols.io ( Denton *et al.*, 2023).

### Sequencing

Pacific Biosciences HiFi circular consensus DNA sequencing libraries were constructed according to the manufacturers’ instructions. DNA sequencing was performed by the Scientific Operations core at the WSI on a Pacific Biosciences SEQUEL II instrument. Hi-C data were also generated from uoEpiScrs1 using the Arima2 kit and sequenced on the Illumina NovaSeq 6000 instrument.

### Genome assembly and curation

Assembly was carried out with Hifiasm ( Cheng *et al.*, 2021) and haplotypic duplication was identified and removed with purge_dups ( Guan *et al.*, 2020). The assembly was then scaffolded with Hi-C data ( Rao *et al.*, 2014) using YaHS ( Zhou *et al.*, 2023). The assembly was checked for contamination and corrected using the gEVAL system ( Chow *et al.*, 2016) as described previously ( Howe *et al.*, 2021). Manual curation was performed using gEVAL, HiGlass ( Kerpedjiev *et al.*, 2018) and Pretext ( Harry, 2022). The mitochondrial and plastid genomes were assembled using MitoHiFi ( Uliano-Silva *et al.*, 2023), which runs MitoFinder ( Allio *et al.*, 2020) or MITOS ( Bernt *et al.*, 2013) and uses these annotations to select the final mitochondrial and plastid contigs, and to ensure the general quality of the sequence.

The assembly of the endosymbiont uoEpiScrs1.Cyanobacterium_sp_1.1 was produced using the following pipeline: to identify cyanobacterial reads, BLAST was run of the PacBio HiFi reads of the uoEpiScrs1 sample against NCBI sequence NZ_AP018341.1 (cyanobacterium endosymbiont of *Rhopalodia gibberula* isolate RgSB Namiki Park) with settings "outfmt 6 -max_target_seqs 10 -max_hsps 1 -evalue 1e-25 -dust yes -lcase_masking". The tool seqkit 2.2.0 was used to isolate reads yielding BLAST hits. These reads were then assembled with Flye 2.9-b1768 with the following settings: --pacbio-hifi --meta --scaffold --keep-haplotypes. prokka 1.14.6 was used to annotate the contigs. NCBI BLAST against the nt database was run with the 16S rRNA gene sequences from this bacterial assembly. The only circular contig in the output of the assembler (contig_39) was identified as cyanobacterial (top BLAST match: "Cyanobacterium endosymbiont of *Rhopalodia gibberula* DNA, isolate: RgSB”). BUSCO 5.2.2 with bacteria_odb10 lineage was run with this contig. The annotation was added by the NCBI Prokaryotic Genome Annotation Pipeline.

The metagenome assembly was generated using metaMDBG and binned using MetaBAT2 (version 2.15-15-gd6ea400), MaxBin (version 2.7), bin3C (version 0.3.3), and MetaTOR. The resulting bin sets of each binning algorithm were individually optimized using DAS Tool (version 1.1.5) and then collectively refined using MAGScoT (version 1.0.0). PROKKA (version 1.14.5) was used to identify tRNAs and rRNAs in each bin, CheckM (version 1.2.1; checkM_DB (release 2015-01-16)) was used to assess bin completeness/contamination, and GTDB-TK (version 2.3.2; GTDB (release 214)) was used to taxonomically classify bins. Taxonomic replicate bins were identified using dRep (version 3.4.0). The final bin set was filtered for bacteria and archaea excluding the previously identified cyanobacteria. ‘MAGs’ were categorised as bins with contamination ≤ 5%, identified 5S, 16S, and 23S rRNA genes along with at least 18 unique tRNAs, and either ≥ 90% completeness or ≥ 50% completeness plus fully circularised chromosomes. Remaining bins with ≤ 10% contamination and ≥ 50% completeness and ‘MAGs’ identified as taxonomic replicates were categorised as ‘binned metagenomes’.

Table 4 contains a list of relevant software tool versions and sources.

**Table 4.** Software tools: versions and sources.

Software tool	Version	Source
bin3C	0.3.3	https://github.com/cerebis/bin3C
BlobToolKit	4.0.7	https://github.com/blobtoolkit/blobtoolkit
BUSCO	5.3.2	https://gitlab.com/ezlab/busco
checkM	2015-01-16	https://ecogenomics.github.io/CheckM/
DAS Tool	1.1.5	https://github.com/cmks/DAS_Tool
dRep	3.4.0	https://github.com/MrOlm/drep
GTDB-Tk	1.2.1	https://github.com/Ecogenomics/GTDBTk
Hifiasm	0.16.1-r375	https://github.com/chhylp123/hifiasm
HiGlass	1.11.6	https://github.com/higlass/higlass
MAGScoT	1.0.0	https://github.com/ikmb/MAGScoT
MaxBin	2.2.7	https://sourceforge.net/projects/maxbin/
Merqury	MerquryFK	https://github.com/thegenemyers/MERQURY.FK
MetaBAT2	2.15-15-gd6ea400	https://bitbucket.org/berkeleylab/metabat
metaMDBG	Pre-release	https://github.com/GaetanBenoitDev/metaMDBG
metaTOR	Pre-release	https://github.com/koszullab/metaTOR
MitoHiFi	2	https://github.com/marcelauliano/MitoHiFi
PretextView	0.2	https://github.com/wtsi-hpag/PretextView
Prokka	1.14.5	https://github.com/tseemann/prokka
purge_dups	1.2.3	https://github.com/dfguan/purge_dups
sanger-tol/genomenote	v1.0	https://github.com/sanger-tol/genomenote
sanger-tol/readmapping	1.1.0	https://github.com/sanger-tol/readmapping/tree/1.1.0
YaHS	yahs-1.1.91eebc2	https://github.com/c-zhou/yahs

### Evaluation of the final assembly

A Hi-C map for the final *E. pelagica* assembly was produced using bwa-mem2 ( Vasimuddin *et al.*, 2019) in the Cooler file format ( Abdennur & Mirny, 2020). To assess the assembly metrics, the *k*-mer completeness and QV consensus quality values were calculated in Merqury ( Rhie *et al.*, 2020). This work was done using Nextflow ( Di Tommaso *et al.*, 2017) DSL2 pipelines “sanger-tol/readmapping” ( Surana *et al.*, 2023a) and “sanger-tol/genomenote” ( Surana *et al.*, 2023b). The genome was analysed within the BlobToolKit environment ( Challis *et al.*, 2020) and BUSCO scores ( Manni *et al.*, 2021; Simão *et al.*, 2015) were calculated.

### Wellcome Sanger Institute – Legal and Governance

The materials that have contributed to this genome note have been supplied by a Tree of Life collaborator. The Wellcome Sanger Institute employs a process whereby due diligence is carried out proportionate to the nature of the materials themselves, and the circumstances under which they have been/are to be collected and provided for use. The purpose of this is to address and mitigate any potential legal and/or ethical implications of receipt and use of the materials as part of the research project, and to ensure that in doing so we align with best practice wherever possible. The overarching areas of consideration are:

• Ethical review of provenance and sourcing of the material

• Legality of collection, transfer and use (national and international)

Each transfer of samples is undertaken according to a Research Collaboration Agreement or Material Transfer Agreement entered into by the Tree of Life collaborator, Genome Research Limited (operating as the Wellcome Sanger Institute) and in some circumstances other Tree of Life collaborators.

## Data Availability

European Nucleotide Archive: *Epithemia pelagica* strain UHM3201 (pennate diatom). Accession number PRJEB54946; https://identifiers.org/ena.embl/PRJEB54946 ( Wellcome Sanger Institute, 2022). The genome sequence is released openly for reuse. The *Epithemia pelagica* strain UHM3201 genome sequencing initiative is part of the Darwin Tree of Life (DToL) project. All raw sequence data and assemblies have been deposited in INSDC databases. The genomes will be annotated using available RNA-Seq data and presented through the Ensembl pipeline at the European Bioinformatics Institute. Raw data and assembly accession identifiers are reported in Table 1.

## Author information

Members of the Wellcome Sanger Institute Tree of Life Management, Samples and Laboratory Team are listed here: https://doi.org/10.5281/zenodo.10066175.

Members of Wellcome Sanger Institute Scientific Operations: Sequencing Operations are listed here: https://doi.org/10.5281/zenodo.10043364.

Members of the Wellcome Sanger Institute Tree of Life Core Informatics team are listed here: https://doi.org/10.5281/zenodo.10066637.

Members of the European Bioinformatics Institute ASG Data Portal team are listed here: https://doi.org//10.5281/zenodo.10076466.

Members of the Wellcome Sanger Institute/Aquatic Symbiosis Genomics Project Leadership are listed here: https://doi.org/10.5281/zenodo.10184833.
