# Supplementary material for: The genome sequences of the marine diatom Epithemia pelagica strain UHM3201 (Schvarcz, Stancheva & Steward, 2022) and its nitrogen-fixing, endosymbiotic cyanobacterium
Source: Wellcome Open Res. 2024 Apr 29;9:232. [Version 1] doi: 10.12688/wellcomeopenres.21534.1 (PMC11167328; doi:10.12688/wellcomeopenres.21534.1)
Supplement: Peer review history

# Reviewer report: Reviewer response for version 1

**DOI:** 10.21956/wellcomeopenres.23805.r84997

**Published:** 2024-06-18

**Authors:** Jean-Marc Aury

**Affiliations:** 1 Génomique Métabolique, Genoscope, Institut François Jacob, CEA, CNRS, Univ Evry, Université Paris-Saclay, Evry, 91057, Évry, France

This study by Schvarcz et al. describes the genome assembly of the diatom *Epithemia pelagica* and its endosymbiotic bacteria. The methods used are relatively standard and common to other projects dedicated to biodiversity sequencing. The genome sequence largely meets the EBP quality standards. Congratulations.

 My main remark is the lack of information concerning the management of other organisms present in the initial sample. Indeed, the assembly performed with Hifiasm allowed the assembly of the *Epithemia pelagica* genome, but also, I imagine, those of other bacteria present in the sample. However, it seems that the authors performed three successive assemblies: one for the host, one for the endosymbiont bacterium, and a final one for the other organisms (metagenome). I think it would have been interesting to describe what the initial assembly contains and why the authors had to use this three-step approach. For example, figures 3 and 4 are based on the cleaned assembly, which ultimately presents limited interest.

 Other minor comments:

- I downloaded the assembly using the following accession number GCA_946965045.2 and generated basic statistics. I found minor differences with the numbers in the article: 18 scaffolds instead of 21 and a size of 60.4 Mb instead of 60.3 Mb. Can the authors explain these discrepancies?
- Please add a citation: "While this new diatom species has yet to be reported elsewhere, metagenomic analyses have detected gene sequences matching E. pelagica’s symbiont in tropical and subtropical marine habitats across the globe, suggesting this symbiosis is more widespread than currently reported."
- What do the authors mean by "manually assembled"?: "The genome of the cyanobacterial endosymbiont of E. pelagica (Chroococcales: Aphanothecaceae) was manually assembled..."
- The authors used the stramenopiles BUSCO dataset, but it contains fewer genes than the eukaryotic one (100 vs 255). Is there a specific reason for this choice?

Are sufficient details of methods and materials provided to allow replication by others?

Yes

Is the rationale for creating the dataset(s) clearly described?

Yes

Are the datasets clearly presented in a useable and accessible format?

Yes

Are the protocols appropriate and is the work technically sound?

Yes

Reviewer Expertise:

Genome assembly, comparative genomics

I confirm that I have read this submission and believe that I have an appropriate level of expertise to confirm that it is of an acceptable scientific standard, however I have significant reservations, as outlined above.

# Reviewer report: Reviewer response for version 1

**DOI:** 10.21956/wellcomeopenres.23805.r85483

**Published:** 2024-06-11

**Authors:** Ruiqi Li

**Affiliations:** 1 University of Colorado Boulder, Boulder, Colorado, USA

Adkins et al. presented a high-quality genome of Epithemia pelagica and its endosymbionts, valuable for studying diatom-cyanobacteria symbiosis. I have a few minor comments:

 1. Citation: For the claim “metagenomic analyses have detected gene sequences matching E. pelagica’s symbiont in tropical and subtropical marine habitats across the globe,” include a citation.

 2. Introduction: The introduction lacks an in-depth discussion of the genome’s importance. Consider citing Coale et al. (2024) for context.

 Coale, Tyler H., et al. "Nitrogen-fixing organelle in a marine alga." Science 384.6692 (2024): 217-222.

Are sufficient details of methods and materials provided to allow replication by others?

Yes

Is the rationale for creating the dataset(s) clearly described?

Yes

Are the datasets clearly presented in a useable and accessible format?

Yes

Are the protocols appropriate and is the work technically sound?

Yes

Reviewer Expertise:

Genomics, Symbiosis

I confirm that I have read this submission and believe that I have an appropriate level of expertise to confirm that it is of an acceptable scientific standard.

# Reviewer report: Reviewer response for version 1

**DOI:** 10.21956/wellcomeopenres.23805.r82798

**Published:** 2024-06-08

**Authors:** Sonia Andrade

**Affiliations:** 1 Universidade de Sao Paulo, São Paulo, State of São Paulo, Brazil

The data  note on genome sequencing of a marine diatom is well presented and soundly analyzed. Besides the genome description, the authors also provided the metagenomes using the "bin" strategy. As data report, there is no discussion or an evolutive perspective, which is expected. The only (very minor) flaw is  that the metagenome results could also bring some information on the core genes found, for example. This information is also descriptive and should have been added to the metagenome description.

Are sufficient details of methods and materials provided to allow replication by others?

Yes

Is the rationale for creating the dataset(s) clearly described?

Yes

Are the datasets clearly presented in a useable and accessible format?

Yes

Are the protocols appropriate and is the work technically sound?

Yes

Reviewer Expertise:

Evolutive biology

I confirm that I have read this submission and believe that I have an appropriate level of expertise to confirm that it is of an acceptable scientific standard.
